# Beta Interferon 1a and Laquinimod Differentially Affect Coagulation-Related Gene Expression in Multiple Sclerosis Patients: Implications for Clinical Efficacy and Side Effects

**DOI:** 10.3390/ijms262211106

**Published:** 2025-11-17

**Authors:** Michael Gurevich, Rina Zilkha-Falb, Joab Chapman, Tali Drori

**Affiliations:** 1 Multiple Sclerosis Center, Sheba Medical Center, Ramat-Gan 52621, Israel; rina.falb@sheba.health.gov.il (R.Z.-F.); joab.chapman@sheba.health.gov.il (J.C.); tali.drori@sheba.health.gov.il (T.D.); 2 Department of Neurology, Gray Faculty of Medical and Health Sciences, Tel-Aviv University, Tel Aviv 6997801, Israel

**Keywords:** multiple sclerosis, immunomodulatory treatment, coagulation

## Abstract

Laquinimod (LAQ) and interferon-β1a (IFN-β1a, Rebif) are immunomodulatory therapies for relapsing–remitting multiple sclerosis (RRMS) that may also influence vascular and hemostatic pathways. This study investigated transcriptional regulation of coagulation- and fibrinolysis-related genes in peripheral blood mononuclear cells (PBMCs) from LAQ- and IFN-β1a–treated RRMS patients. In the LAQ cohort, within-subject paired analysis revealed significant downregulation of procoagulant and antifibrinolytic transcripts, including F2, F10, SERPINE1 (PAI-1), and TFPI, after six months of treatment (FDR < 0.01, |fold change| ≥ 1.5). These findings suggest that LAQ exerts an anticoagulant and antifibrinolytic transcriptional effect, potentially mediated through aryl hydrocarbon receptor (AhR) signaling. Western blot analysis confirmed decreased PAI-1 protein expression. In contrast, IFN-β1a treatment induced a distinct transcriptional pattern. Genes associated with fibrinolysis and endothelial stabilization (THBD, ANXA2, SERPINA1) were upregulated, while procoagulant mediators (F2R, F13A1, PROS1) were downregulated. Correlation analysis demonstrated significant relationships between interferon-inducible genes and coagulation-related transcripts, suggesting coordinated regulation between type I interferon signaling and vascular pathways. Collectively, these results indicate that LAQ and IFN-β1a exert opposing yet convergent influences on coagulation and fibrinolytic networks in immune cells. Both therapies modulate transcriptional regulators of vascular homeostasis, revealing a potential molecular interface between immune modulation and hemostatic balance in RRMS. These findings are exploratory and hypothesis-generating, warranting further functional and clinical validation.

## 1. Introduction

Laquinimod (LAQ) is an oral immunomodulatory agent developed for the treatment of relapsing–remitting multiple sclerosis (RRMS). It acts on both peripheral immune cells and central nervous system (CNS)-resident cells, exerting its effects through modulation of microglial and astrocytic activation and by enhancing the production of brain-derived neurotrophic factor (BDNF) [1,2,3,4]. Mechanistically, LAQ has been shown to activate the aryl hydrocarbon receptor (AhR), a key regulator of immune tolerance and inflammation [5]. Clinical trials have demonstrated modest effects of LAQ on relapse rate and magnetic resonance imaging (MRI) outcomes in RRMS, with evidence of brain volume preservation [6,7,8,9,10]. In the phase 3 CONCERTO trial, the higher dose of LAQ (1.2 mg/day) was associated with an increased incidence of serious cardiovascular adverse events, including myocardial infarction, leading to the early discontinuation of that treatment arm [11]. Although no such events were reported with the 0.6 mg dose, these findings raised questions about the potential vascular or hemostatic effects of LAQ that warrant further investigation.

In parallel, interferon beta-1a (IFN-β1a), another first-line treatment for RRMS, modulates immune function through type I interferon signaling. IFN-β1a, marketed as Rebif, induces a broad set of interferon-stimulated genes (ISGs), modulates cytokine balance, and reduces T-cell activation and trafficking into the CNS [12,13,14,15,16]. However, emerging evidence suggests that type I interferons can also influence coagulation pathways. Specifically, interferon signaling has been linked to prothrombotic effects via induction of tissue factor, plasminogen activator inhibitor-1 (PAI-1), and other mediators involved in clot formation [17,18]. In MS patients, recombinant type I interferon therapies have been reported to increase the risk of thrombotic microangiopathies [19]. A recent review by Ryan and O’Neill (2023) highlighted the growing recognition of type I interferons as regulators of coagulation, with implications for both autoimmune pathology and vascular risk [20]. Given that MS therapies may differ not only in their immune targets but also in systemic effects, including coagulation, a comparative analysis is timely.

In this study, we investigated the transcriptional effects of LAQ and IFN-β1a (Rebif) on coagulation-related genes in RRMS patients. Our aim was to determine whether these two immunomodulatory agents exert opposing or distinct effects on coagulation signaling and whether such regulation could have implications for therapeutic mechanisms or potential cardiovascular risk.

## 2. Results

### 2.1. LAQ Cohort

Comprehensive transcriptomic profiling of PBMCs from RRMS patients treated with LAQ was previously reported as part of a substudy of the ALLEGRO clinical trial [21]. In brief, 25 patients were enrolled (mean age 38.0 ± 2.0 years; female/male ratio 16/9), including 13 in the LAQ treatment arm (mean age 38.8 ± 2.3 years; median EDSS 1.5 [0.5–2.5]; 8 females, 5 males) and 12 in the placebo group (mean age 37.2 ± 3.4 years; 8 females, 4 males). Peripheral blood samples were obtained at baseline, one month, and six months after treatment initiation. Treatment with LAQ induced broad, time-dependent transcriptional changes: after one month of therapy, 354 genes were significantly modulated, and by six months, the number of most informative genes (MIGs) increased to 1562. The complete list of MIGs associated with the global LAQ response was reported previously [21] and is not repeated here to maintain the focus of the current analysis on coagulation-related molecular mechanisms.

### 2.2. Modulation of Coagulation-Related Genes by LAQ

**Figure 1:**
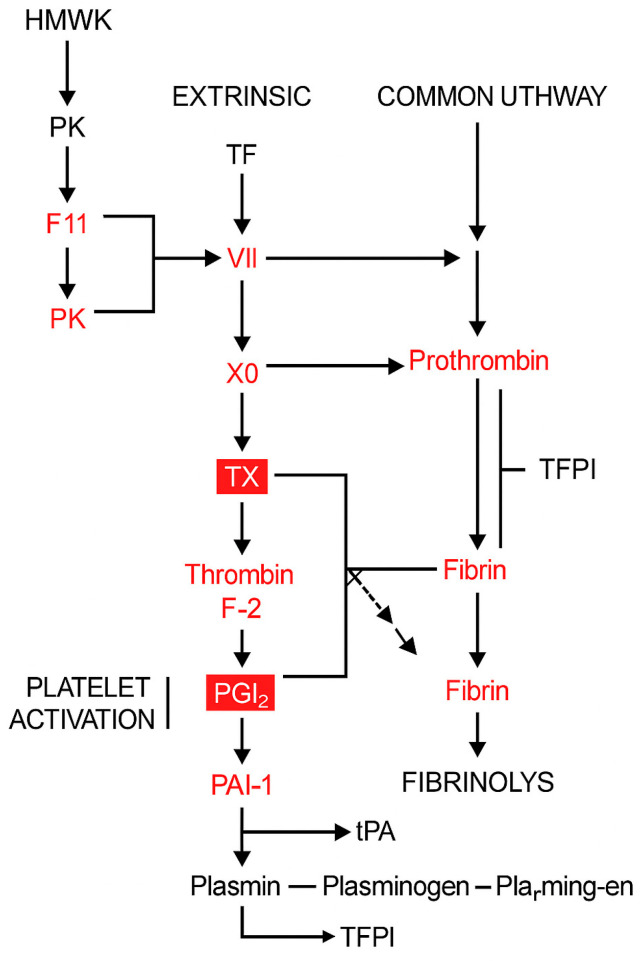
Schematic representation of the coagulation and fibrinolysis pathways, highlighting genes downregulated by LAQ. This figure illustrates the intrinsic, extrinsic, and common pathways of coagulation, as well as the fibrinolytic system and platelet activation. Genes shown in red represent those found to be significantly downregulated in PBMCs from RRMS patients following treatment with LAQ (0.6 mg/day) for 6 months. This transcriptional suppression suggests that LAQ reduces the activity of several critical regulators of coagulation and fibrin turnover. Abbreviations: F2—Prothrombin (precursor to thrombin), F11 (XI)—Factor XI (intrinsic pathway), F7 (VII)—Factor VII (extrinsic pathway), TF—Tissue Factor (initiates extrinsic pathway), PK—Prekallikrein, HMWK—High-molecular-weight kininogen, TX—Thromboxane. Red boxed elements highlight critical regulatory hubs within the pathway that are both affected and central to pathway integration (e.g., F2 [thrombin], PAI-1), based on their biological importance and number of downstream effects. Black arrows: normal activation flow in the pathway. Flat-ended lines: Inhibitory interactions.

Among the 354 and 1552 most informative genes (MIGs) altered after one and six months of LAQ treatment, respectively, 3 and 11 genes were identified as associated with coagulation or fibrinolysis pathways based on Gene Ontology and Reactome annotations (Table 1). These included genes involved in thrombin generation (F2, F10, F11), fibrin clot formation (FGA, ITGA2B/ITGB3, encoding GPIIb/IIIa*), and fibrinolytic regulation (PLAT, SERPINE1 [PAI-1], PRSS1, PRSS3), all of which were significantly downregulated after six months of treatment (*p* < 0.01). Among these, three genes—F2, BDKRB2, and PROS1—were already downregulated after one month, indicating an early transcriptional impact of LAQ on coagulation-related pathways. Taken together, these results suggest that LAQ modulates transcriptional regulators of clotting and fibrinolysis in circulating immune cells (Figure 1).

**Figure 2:**
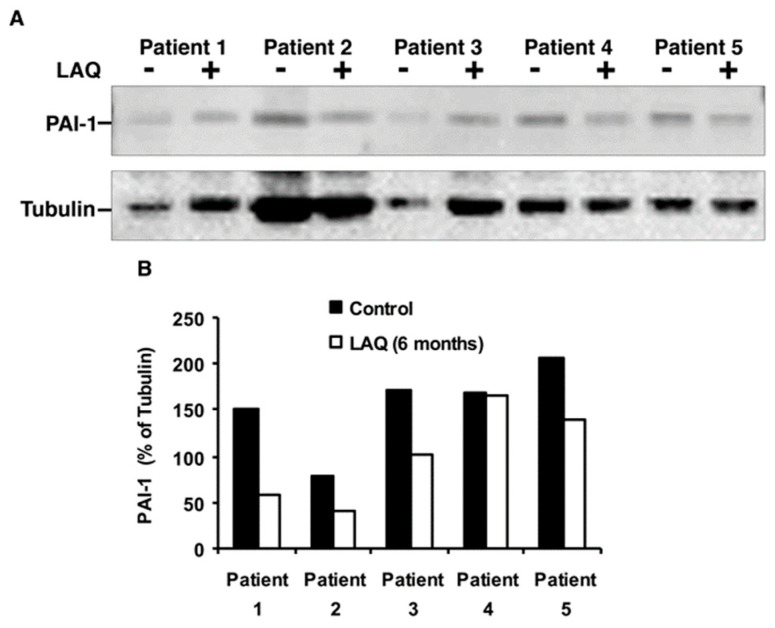
Expression of PAI-1 (SERPINE1) in PBMCs of RRMS patients treated with LAQ. (**A**) Representative cropped blots showing PAI-1 and α-Tubulin before (-) and after (+) six months of treatment (*n* = 5 paired samples, 30 µg protein per lane). (**B**) Quantification of relative PAI-1 levels normalized to tubulin; *p* = 0.02, paired *t*-test.

Importantly, in accordance with our gene expression results, which show significant downregulation of the key PAI-1 gene, the verification by Western blot analysis shown in Figure 2, demonstrates reduced expression by 30–50% of PAI-1 protein in four out of five patients after 6 months of LAQ treatment (*p* = 0.02, paired *t*-test, *n* = 5). These results suggest that LAQ down modulates PAI-1 and other coagulation factors and thereby enables tPA activity and fibrinolysis that are associated with reduced clinical severity in mouse model of MS [22]. Representative cropped blots are shown in Figure 2.

### 2.3. Rebif Cohort 1: Modulation of Coagulation-Related Genes by IFN-β1a (Rebif)

To assess whether Rebif modulates coagulation-related transcriptional programs, we analyzed PBMC gene-expression profiles from 12 RRMS patients (mean age 41.0 ± 3.1 years; 7 females and 5 males; median EDSS = 2.0 [0.5–3.0]) before and after three months of treatment with Rebif (44 µg subcutaneously, three times per week). Differential-expression analysis identified 1101 significant most informative genes (MIGs). Functional enrichment analysis revealed strong overrepresentation of the Type I Interferon Signaling Pathway (*p* = 1.2 × 10^−10^), consistent with the known mechanism of action of IFN-β therapies, as well as the Regulation of Immune Response pathway (*p* = 3.4 × 10^−9^), highlighting modulation of immune activity. Although global enrichment did not identify coagulation pathways as significantly overrepresented in the Rebif cohort, several individual genes involved in hemostasis and vascular regulation were modulated. In total, 18 coagulation-associated genes were significantly altered following Rebif treatment (Table 2).

Several of the identified genes (e.g., F2, TFPI, SERPINE1, PLAT, PROS1, F2R) represent central regulatory hubs within the coagulation system. The inclusion of these well-characterized and mechanistically important genes strengthens our hypothesis. Specifically, the upregulated anticoagulant and anti-inflammatory genes included SERPINA1 (alpha-1 antitrypsin), a serine protease inhibitor; THBD (thrombomodulin), a cofactor in protein C activation; and ANXA2 (annexin A2), a mediator of fibrinolysis. Additional upregulated genes—RHOB (Ras homolog family member B), ARHGEF3 (Rho guanine nucleotide exchange factor 3), MAPK11, and EGFR (epidermal growth factor receptor)—are signaling intermediates linked to endothelial or platelet biology.

**Figure 3:**
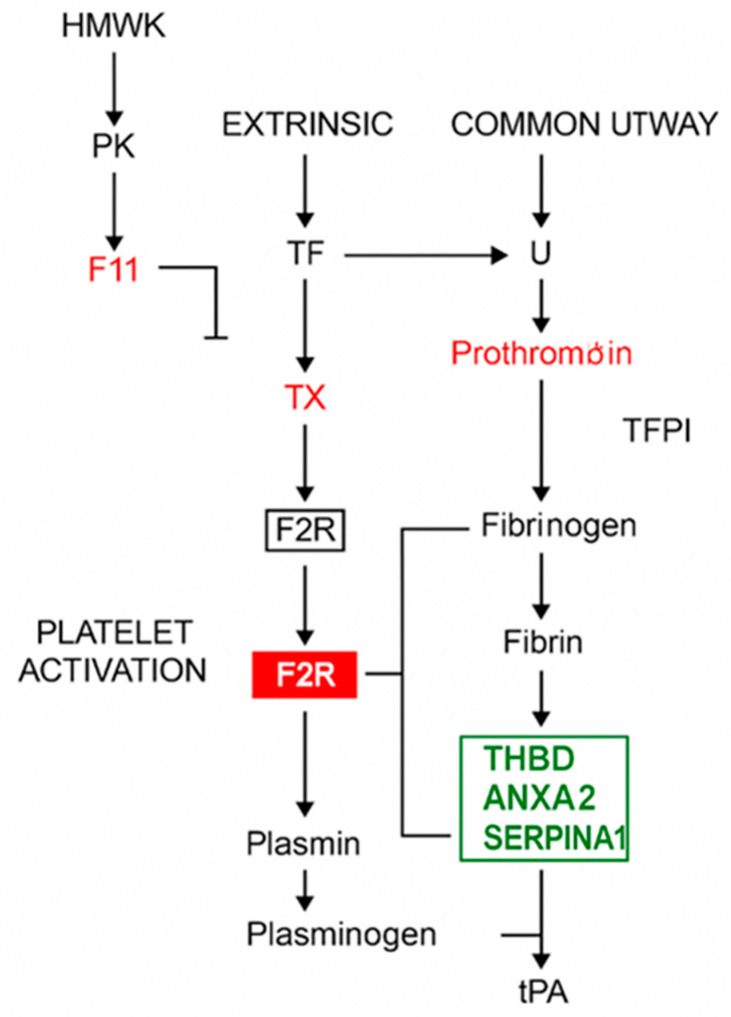
Effect of Rebif on coagulation and fibrinolysis pathways. This schematic represents the classical coagulation cascade (intrinsic, extrinsic, and common pathways), platelet activation, and fibrinolysis. Gene products regulated by IFN-β1a treatment in RRMS patients are highlighted within the pathway. Red box: genes significantly downregulated after IFN-β treatment. Green box: Genes significantly upregulated after IFN-β treatment. Black arrows: activation or conversion steps in the cascade, T-bar lines: Inhibitory regulatory actions, red font: additional components known to be influenced downstream. Abbreviations: HMWK—High-molecular-weight kininogen, PK—Prekallikrein, TF—Tissue factor, TX—Factor X (X_a_), activated in the common pathway, F2R—Protease-activated receptor-1 (PAR-1), thrombin receptor, THBD—Thrombomodulin, ANXA2—Annexin A2, SERPINA1—Alpha-1 antitrypsin, tPA—Tissue plasminogen activator, Plasminogen/Plasmin—Enzymes for fibrin degradation.

Conversely, downregulated genes encoded prothrombotic or cytoskeletal regulators, including F2R (thrombin receptor/PAR1), F13A1 (coagulation factor XIII A1 subunit), and PROS1 (protein S), along with cytoskeletal genes such as MYLK and MYL9. These findings suggest that Rebif activates a mixed transcriptional program influencing both pro- and anticoagulant regulators, with a notable reduction in F2R (PAR1), a thrombin receptor implicated in neuroinflammation. The proposed mechanism underlying Rebif’s transcriptional effects is illustrated in Figure 3.

### 2.4. Correlation Between IFN-Stimulated and Coagulation-Related Genes

To explore mechanistic links between type I interferon (IFN) signaling and coagulation-related transcriptional changes, we performed correlation analyses between well-known IFN-stimulated genes (MX1, OAS1, OAS2, IFI44L)—all significantly overexpressed among the MIGs—and coagulation-associated transcripts. Significant positive correlations were observed between OAS1 and several coagulation-related genes, including SERPINA1 (r = 0.94, *p* = 5.77 × 10^−4^) and F2RL2 (r = 0.90, *p* = 3.26 × 10^−3^). Conversely, significant negative correlations were detected between IFI44L and coagulation-related genes such as TFPI (r = −0.90, *p* = 5.38 × 10^−3^) and PLCB2 (r = −0.84, *p* = 2.11 × 10^−2^) (Table 3). These associations support a regulatory interplay between IFN-β–induced gene networks and the coagulation/fibrinolysis balance, reinforcing the hypothesis that IFN-β may indirectly modulate vascular or thrombotic pathways through transcriptional regulation favoring an overall anti-coagulant and pro-fibrinolytic gene-expression pattern.

### 2.5. Rebif Cohort 2: Cross-Sectional Analysis of Rebif-Treated Versus Untreated Patients

An additional assessment of type I IFN treatment effects on coagulation and fibrinolysis pathways was performed by analyzing differentially expressed genes in PBMCs from Rebif-treated and untreated RRMS patients (Rebif cohort 2). The clinical and demographic characteristics of the participants (*n* = 35; mean age 40.2 ± 1.2 years; female/male ratio 20/15; Rebif-treated/untreated 10/25; mean EDSS 2.2 ± 0.2; disease duration 6.8 ± 0.8 years) were previously reported [23]. Comprehensive transcriptome analysis focused on genes annotated to coagulation and fibrinolysis pathways according to Gene Ontology and Reactome databases identified 37 differentially expressed MIGs (Table 4). Notably, the majority of MIGs (83%) identified in the longitudinal analysis of Rebif cohort 1 were also present among the differentially expressed genes in Rebif cohort 2, all showing consistent directional changes—including F2R, RHOB, THBD, and SERPINA1.

## 3. Discussion

In this study, we demonstrate that LAQ treatment in RRMS patients leads to consistent downregulation of genes and proteins involved in the coagulation and fibrinolytic systems. Transcriptomic profiling of PBMCs revealed suppression of coagulation factors (F2, F10, F11, FGA, FGB) and regulators of fibrinolysis such as SERPINE1 (PAI-1), PLAT, and TFPI. These changes were corroborated at the protein level, with Western blot analysis confirming reduced expression of PAI-1 and TGF-β1 after six months of treatment. Together, these results indicate that LAQ induces a systemic anticoagulant and antifibrinolytic transcriptional signature. A comparable anticoagulant effect was observed in patients treated with Rebif; however, in contrast, these patients exhibited increased expression of fibrinolytic pathway components.

Given that LAQ is a known agonist of the aryl hydrocarbon receptor (AhR) [5,24,25], which has been implicated in the transcriptional regulation of vascular and fibrinolytic genes [26], it is plausible that the observed downregulation of coagulation-related transcripts following LAQ treatment is mediated through AhR signaling. Previous studies have linked AhR activation to suppression of SERPINE1, F2, and TFPI, further supporting this mechanistic pathway.

The safety profile of LAQ at the 0.6 mg daily dose has been extensively evaluated in the phase III ALLEGRO and BRAVO clinical trials, which together enrolled nearly 2000 patients with RRMS. Pooled safety data from these studies indicate that LAQ is generally well tolerated, with predominantly mild, transient, and reversible adverse events [9]. Importantly, no increased incidence of cardiovascular events, infections, or malignancies was observed compared with placebo. Laboratory abnormalities such as elevated liver enzymes, mild anemia, and increased fibrinogen levels typically appeared within the first 90 days and resolved or stabilized during continued treatment without clinical sequelae. These findings reinforce the clinical manageability of the 0.6 mg dose, particularly in light of earlier concerns regarding cardiovascular risks associated with higher doses or related compounds such as roquinimex. Notably, fibrinogen elevations were modest and not associated with thrombotic events, consistent with the transcriptional downregulation of coagulation-related genes observed in our dataset.

While suppression of coagulation pathways might imply a reduced thrombotic risk, the cardiovascular events reported at higher LAQ doses suggest a more complex interaction. One possibility is that antifibrinolytic effects outweigh the protective anticoagulant effects, leading to increased thrombotic risk. Alternatively, chronic inhibition of coagulation and fibrinolytic regulators could provoke compensatory prothrombotic responses upon discontinuation or dose escalation, potentially resulting in rebound hypercoagulability—phenomena described with other anticoagulant therapies [27,28].

TGF-β1, a cytokine with dual roles in immune regulation and vascular integrity, is essential for maintaining endothelial function. Suppression of TGF-β1 signaling, as demonstrated in our previous work [21], could impair endothelial stability and promote vascular injury. Studies have shown that TGF-β1 supports endothelial homeostasis by regulating cell proliferation, migration, and extracellular matrix production [29,30,31].

PAI-1 (plasminogen activator inhibitor-1) also plays a central role in fibrinolysis and vascular remodeling. Although elevated PAI-1 is a risk factor for thrombosis and cardiovascular disease [32,33], excessively reduced levels may impair vascular repair and destabilize plaques, particularly under inflammatory conditions [34]. Thus, LAQ-mediated suppression of PAI-1 could disturb the delicate balance between thrombus formation and resolution.

The present transcriptomic findings suggest that LAQ modulates pathways involved in coagulation, fibrinolysis, and vascular regulation. While these transcriptional changes conceptually align with clinical observations from the CONCERTO trial at higher doses (1.2 mg) [11], the current study was not designed to assess safety or clinical outcomes, and no causal inference can be made. Therefore, these data should be interpreted as exploratory and hypothesis-generating, offering preliminary mechanistic insights rather than clinical validation. Future prospective studies integrating transcriptomic, proteomic, and functional assays are needed to determine whether these molecular changes have predictive or translational relevance to treatment safety or efficacy.

Because PBMCs do not include platelets or endothelial cells that directly mediate thrombosis and fibrinolysis, the observed transcriptional changes may partially reflect shared regulatory mechanisms across immune-cell subsets. Altogether, our findings suggest that LAQ influences key regulators of the coagulation system in a manner that could contribute both to its beneficial CNS effects and to potential systemic risks at higher doses. Further studies are required to clarify the in vivo hemostatic consequences of these molecular changes and to determine whether coagulation-related biomarkers could assist in risk stratification of LAQ-treated patients.

Rebif (IFN-β1a), a well-established first-line immunomodulatory therapy for RRMS, exhibited a distinct and partially opposing transcriptional signature compared with LAQ, particularly in genes regulating coagulation and fibrinolysis. In both short-term (3-month) and long-term treated cohorts, we observed consistent upregulation of genes associated with fibrinolysis and endothelial stability, including SERPINA1 (alpha-1 antitrypsin), THBD (thrombomodulin), and ANXA2 (annexin A2) [35,36,37]. These genes have been implicated in promoting anticoagulant and antithrombotic pathways. Conversely, downregulation of procoagulant regulators such as F2R (PAR1), F13A1, and PROS1 suggests that Rebif may also attenuate thrombin responsiveness and fibrin crosslinking [38,39,40]. This mixed regulatory profile—stabilizing yet modulating the coagulation system—may reflect a broader vascular and immune regulatory role of IFN-β therapy.

To explore potential mechanistic links between IFN-β treatment and transcriptional regulation of coagulation genes, we examined correlations between canonical IFN-inducible genes (OAS1, OAS2, MX1, IFI44) and coagulation-related transcripts. The observed significant correlations suggest a coordinated regulation of immune antiviral signaling and thrombotic sensitivity. Specifically, positive correlations were detected between OAS1 and SERPINA1, a serine protease inhibitor with anti-inflammatory and anticoagulant properties. These findings imply that type I interferon-induced gene networks may function as upstream modulators of select coagulation genes in peripheral immune cells. This interpretation aligns with emerging evidence positioning IFN-I signaling as a regulatory axis in vascular biology and platelet–endothelial homeostasis, particularly under inflammatory conditions [18,20,41]. Collectively, these results support the hypothesis that transcriptional changes in coagulation pathways after IFN-β therapy may, at least in part, arise secondary to IFN-stimulated gene activation.

The transcriptional modulation of coagulation and fibrinolytic pathways by IFN-β1a may also have clinical relevance, particularly regarding vascular health in MS patients. Although IFN-β therapies are generally well tolerated, some studies have reported associations with vascular events. For example, a nested case–control study in British Columbia identified a 1.8-fold increased risk of stroke among IFN-β–treated patients compared with untreated individuals [42]. The biological plausibility of this association is supported by evidence that IFN-β can alter endothelial function and enhance platelet adhesion, potentially contributing to vascular events. However, such risks remain relatively low and must be weighed against the well-established benefits of IFN-β in reducing relapse rates and delaying disability progression in RRMS. The observed transcriptional changes in coagulation-related genes may therefore represent a complex interplay between IFN-β’s immunomodulatory effects and vascular homeostasis, warranting further investigation. Another possible explanation for prothrombotic effects is the induction of Th2-mediated autoimmune conditions such as antiphospholipid syndrome and lupus, both recognized complications of IFN-β therapy [43].

This study has several limitations. Adjustment for PBMC cell-type composition was not feasible, and deconvolution analysis could not be performed; therefore, part of the observed transcriptional variation—particularly in platelet- or endothelial-associated genes—may reflect differences in immune-cell proportions rather than direct regulation of coagulation pathways. The limited sample size of the Rebif cohort constrains the precision of correlation analyses, which should be considered exploratory and hypothesis-generating. Independent qRT-PCR validation was not possible because the original trial samples are no longer available; however, the paired within-subject design provides strong internal consistency, and key genes (SERPINE1, F2, TFPI, PROS1) showed reproducible regulation in prior studies, supporting the robustness of these findings.

In summary, this study demonstrates that both LAQ and IFN-β (Rebif) treatments modulate transcriptional networks related to coagulation, fibrinolysis, and vascular regulation, suggesting a molecular interface between immune and hemostatic pathways in multiple sclerosis. In LAQ-treated patients, coordinated downregulation of coagulation-related transcripts may reflect dose-dependent immune–vascular effects, whereas IFN-β-associated correlations between interferon-inducible and coagulation genes support shared mechanisms. These findings are exploratory and hypothesis-generating, providing preliminary molecular insights rather than causal or prognostic evidence. Further prospective and functional studies are required to confirm the biological relevance and potential clinical implications of these transcriptional changes.

## 4. Materials and Methods

### 4.1. Study Design and Participants

This was a retrospective, exploratory study designed to evaluate transcriptional changes in genes associated with coagulation pathways in patients with relapsing–remitting multiple sclerosis (RRMS) treated with either LAQ or Rebif. Participants were recruited at the Multiple Sclerosis Center, Sheba Medical Center. Peripheral blood samples were collected longitudinally at three time points: prior to treatment (baseline) and at one and six months after initiation of LAQ (LAQ cohort); and at baseline and three months after initiation of Rebif (Rebif cohort 1). In addition, a cross-sectional comparison was performed in a separate group of RRMS patients receiving Rebif treatment prior to enrollment versus age- and sex-matched untreated RRMS patients (Rebif cohort 2).

**Figure 4:**
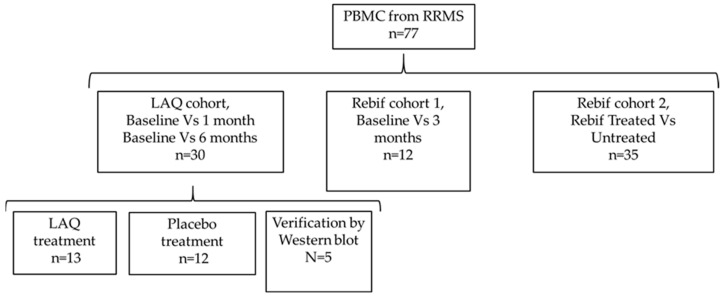
Study design.

The LAQ and placebo groups were randomized and balanced for baseline demographic and clinical characteristics as part of the ALLEGRO clinical trial design. This randomization minimized potential confounding by factors such as age, sex, and disease duration, allowing subsequent transcriptomic analyses to focus primarily on within-subject treatment effects rather than between-group differences. All participants provided written informed consent, and the study protocol was approved by the Sheba Medical Center Institutional Review Board. The overall study design is illustrated in Figure 4.

Participants were eligible for inclusion if they met all of the following criteria: (a) Diagnosis of RRMS according to the 2010 revised McDonald criteria; (b) age between 18 and 60 years; (c) Expanded Disability Status Scale (EDSS) score between 0.0 and 5.5 at baseline; (d) no immunosuppressive therapy for at least three months prior to enrollment; (e) no corticosteroid treatment within 30 days before sample collection; (f) planned initiation of first-line monotherapy with either LAQ (0.6 mg daily) for the LAQ cohort or Rebif (44 µg subcutaneously, three times weekly) for Rebif cohort 1; (g) for Rebif cohort 2, treatment with Rebif (new formulation, Merck-Serono, Merck KGaA, Darmstadt, Germany) at a dose of 22 or 44 µg, subcutaneously three times per week, for at least four months prior to study initiation; (h) untreated participants were free from immunomodulatory drugs for at least one month prior to sampling; and (i) in Rebif cohort 2, treated participants had received IFN-β therapy for at least four months before enrollment.

Exclusion criteria included pregnancy, breastfeeding, known coagulopathies or thrombotic disorders, active infection at baseline, or any autoimmune or systemic disease affecting immune function. Because both the LAQ and Rebif cohort 1 analyses were based on paired within-subject comparisons, baseline differences between treatment groups were not applicable. In contrast, Rebif cohort 2 was cross-sectional (treated vs. untreated RRMS patients) and did not include baseline samples. As previously reported [23], these groups did not differ significantly in age, sex, EDSS, or disease duration at the time of sampling.

To ensure that the observed transcriptional effects were specific to LAQ, statistically significant expression changes detected in the placebo group with the same directionality were excluded from further analysis, as described previously [21]. Consequently, all downstream analyses and interpretations were based exclusively on LAQ-specific MIGs, representing placebo-adjusted molecular effects.

### 4.2. Sample Processing and RNA Preparation

Peripheral blood mononuclear cells (PBMCs) were isolated from whole blood using Ficoll-Paque density gradient centrifugation. Total RNA was extracted with TRIzol reagent (Invitrogen, Carlsbad, CA, USA), followed by DNase I treatment to remove genomic DNA contamination and purification using Phase Lock Gel columns (Eppendorf, Hamburg, Germany). RNA quantity and integrity were assessed with an Agilent 2100 Bioanalyzer (Agilent Technologies, Sant Clara, CA, USA) to ensure suitability for downstream transcriptomic analysis.

#### 4.2.1. Gene Expression Profiling

Gene expression profiling was performed using Affymetrix Human Genome U133A 2.0 (Santa Clara, CA, USA) microarrays, encompassing approximately 14,500 well-annotated genes. Quality control (QC) procedures followed standard Affymetrix and Partek protocols, including assessment of array-intensity distributions, residual diagnostics, and principal component analysis (PCA). Arrays that did not meet QC criteria were excluded from downstream analysis. Probe-to-gene mapping was based on the Affymetrix HG-U133A-2 annotation file (release 36). Genes associated with coagulation, fibrinolysis, and vascular biology were identified using Gene Ontology (GO), Reactome, and Ingenuity Pathway Analysis (IPA) annotations.

#### 4.2.2. Microarray Analysis

LAQ cohort. Raw Affymetrix HG-U133A-2 CEL files were processed in Partek Genomics Suite (version 7.19.1125) using the Robust Multi-array Average (RMA) algorithm, which includes background correction, quantile normalization, log_2_ transformation, and median-polish summarization. Within-subject changes (baseline vs. one month; baseline vs. six months) were tested using paired *t*-tests, and *p*-values were adjusted across all probe sets using the Benjamini–Hochberg false discovery rate (FDR) procedure. Most informative genes (MIGs) were defined as those with FDR-adjusted *p* < 0.01 and an absolute fold change ≥ 1.5. No additional batch or demographic covariates were applied at the testing stage because the paired design—and randomization within the ALLEGRO trial—minimized between-subject and batch variability.

Rebif cohorts 1 and 2. Raw CEL files were normalized in R/Bioconductor using single-sample microarray normalization. Potential batch effects were corrected using ComBat from the SVA package. Probe-to-gene mapping employed the Affymetrix HG-U133A-2 annotation file (release 36). For Rebif cohort 1, within-subject differences (baseline vs. three months) were assessed by paired *t*-tests in Partek, with multiplicity controlled by Benjamini–Hochberg FDR correction. MIGs were defined as FDR-adjusted *p* < 0.05 and |fold change| ≥ 1.5.

For the Rebif cohort 2, differential expression was analyzed in Partek using a linear model with treatment status as a fixed factor. *p*-values were adjusted for multiple testing using the Benjamini–Hochberg FDR method, and MIGs were defined as FDR-adjusted *p* < 0.05 and |fold change| ≥ 1.5.

Power estimation. For the paired LAQ and Rebif 1 cohorts, sample sizes (*n* = 13 and *n* = 12, respectively) provided >80% power to detect ≥ 1.5-fold expression changes at FDR < 0.01–0.05 for transcripts of moderate variability. The Rebif 2 cross-sectional cohort (*n* = 10 treated, *n* = 25 untreated) had lower within-group precision but sufficient power to detect moderate (≥1.5-fold) differences at FDR < 0.05, consistent with its exploratory design.

Correlation analysis. Pairwise correlations between interferon-inducible and coagulation-related genes were calculated using Pearson’s correlation coefficient (r) on log_2_-transformed expression values. This approach was selected because it allows estimation of 95% confidence intervals for correlation coefficients and facilitates parametric adjustment for multiple testing. *p*-values were adjusted using the Benjamini–Hochberg FDR method applied across all tested pairs.

#### 4.2.3. Protein Validation

For a subset of patients in the LAQ cohort, protein expression of PAI-1 was evaluated via Western blot. Protein samples were prepared from the phenol/ethanol fractions of PBMCs obtained after six months of LAQ treatment and compared with PBMC samples from the same patients before treatment. From each sample, 30 µg of total protein was separated on 10% SDS–polyacrylamide gels (SDS-PAGE). Blots were incubated with rabbit anti–PAI-1 antibody (1:500, sc-8979 Santa Cruz Biotechnology, Inc., Santa Cruz, CA, USA) and mouse anti–tubulin antibody (1:500, sc-5274 Santa Cruz Biotechnology, Inc., Santa Cruz, CA, USA). Appropriate secondary antibodies anti-mouse or anti-rabbit conjugated horseradish peroxidase [(HRP) 1:10,000; Jackson immunoresearch] were used. Blots were analyzed via standard chemiluminescence (Supersignal Kit, Pierce, Rockford, IL, USA) and visualization performed done using a ChemiDoc™ XRS System (Bio-Rad, Hercules, CA, USA). Signal intensities of each protein band and the corresponding background were quantified using Quantity One software (version 4.6.9; Bio-Rad). Background-subtracted protein expression values were normalized to tubulin and plotted as relative protein levels for each patient before and after LAQ treatment.

## Figures and Tables

**Table 1:** LAQ-induced transcriptional regulation of the coagulation mechanism in PBMC.

Gene Symbol	Gene Name	1 Month *p*-Value (FDR)	1 Month Log2FC	6 Months *p*-Value (FDR)	6 Months Log2FC
F2	Coagulation factor II (thrombin)	8.2 × 10^−3^	−1.14	5.4 × 10^−3^	−1.17
BDKRB2	Bradykinin receptor B2	7.0 × 10^−3^	−1.14	_	_
PROS1	Protein S	9.1 × 10^−3^	−1.96		
F10	Coagulation factor X	_	_	4.5 × 10^−3^	−1.16
F11	Coagulation factor XI	_	_	4.4 × 10^−3^	−1.19
FGA	Fibrinogen alpha chain	_	_	6.3 × 10^−3^	−1.11
GPIIb/III3	Fibrinogen receptor	_	_	1.1 × 10^−3^	−1.24
TFPI	Tissue factor pathway inhibitor	_	_	6.4 × 10^−3^	−1.16
CPB2	Carboxypeptidase B2	_	_	4.2 × 10^−3^	−1.13
PLAT	Tissue plasminogen activator	_	_	5.3 × 10^−3^	−1.16
SERPINE1	Plasminogen activator inhibitor-1	_	_	1.8 × 10^−3^	−1.22
PRSS1/3	Serine proteases	_	_	3.6 × 10^−3^	−1.23

**Table 2:** Rebif induced transcriptional regulation of the coagulation mechanism in PBMC.

Gene Symbol	Gene Name	*p* Value (FDR)	Log2FC
RHOB	ras homolog family member B	4.80 × 10^−3^	1.33
SERPINA1	serpin peptidase inhibitor, clade A, member 1	1.80 × 10^−3^	1.23
ANXA2	annexin A2	3.20 × 10^−3^	1.12
ITPK1	inositol-tetrakisphosphate 1-kinase	7.00 × 10^−3^	1.07
ARHGEF3	Rho guanine nucleotide exchange factor (GEF) 3	2.20 × 10^−3^	1.07
THBD	thrombomodulin	1.70 × 10^−3^	1.1
NRAS	neuroblastoma RAS viral (v-ras) oncogene homolog	9.40 × 10^−3^	1.05
EGFR	epidermal growth factor receptor	1.00 × 10^−2^	1.03
MAPK11	mitogen-activated protein kinase 11	1.70 × 10^−3^	1
PLCB2	phospholipase C, beta 2	2.00 × 10^−3^	1.02
HNRNPA1	heterogeneous nuclear ribonucleoprotein A1	1.20 × 10^−2^	−1.1
GNG7	guanine nucleotide-binding protein (G protein), gamma 7	9.70 × 10^−4^	1.07
F2R	coagulation factor II (thrombin) receptor (PAR1)	1.60 × 10^−2^	−1.1
EGF	epidermal growth factor	8.50 × 10^−3^	−1.1
PROS1	protein S (alpha)	7.40 × 10^−3^	−1.18
MYLK	myosin light chain kinase	3.90 × 10^−3^	−1.24
MYL9	myosin, light chain 9, regulatory	5.60 × 10^−3^	−1.33
F13A1	coagulation factor XIII, A1 polypeptide	8.13 × 10^−3^	−1.39

**Table 3:** Correlation between PCMC transcriptional changes of main IFN-inducible MIGs and coagulation-related MIGs after 3 months of Rebif treatment.

Gene Symbol	r	Lower CI	Upper CI	*p*-Value	*p*-Value (FDR)	Gene Title
**Correlation with OAS1**						
SRC	0.96	0.87	0.99	5.59 × 10^−7^	1.03 × 10^−4^	SRC proto-oncogene
SERPINA1	0.94	0.79	0.98	6.24 × 10^−6^	5.77 × 10^−4^	serpin peptidase inhibitor, clade A, member 1
F2RL2	0.90	0.67	0.97	7.46 × 10^−5^	3.26 × 10^−3^	coagulation factor II (thrombin) receptor-like 2
CREB1	0.89	0.66	0.97	8.61 × 10^−5^	3.26 × 10^−3^	cAMP-responsive element binding protein 1
MAPK1	0.89	0.66	0.97	8.80 × 10^−5^	3.26 × 10^−3^	mitogen-activated protein kinase 1
ANXA2	0.87	0.59	0.96	2.47 × 10^−4^	7.63 × 10^−3^	annexin A2
RHOC	0.83	0.49	0.95	8.00 × 10^−4^	1.88 × 10^−2^	ras homolog family member C
CREB1	0.83	0.48	0.95	8.90 × 10^−4^	1.88 × 10^−2^	cAMP-responsive element binding protein 1
NRAS	0.83	0.48	0.95	9.14 × 10^−4^	1.88 × 10^−2^	neuroblastoma RAS viral (v-ras) oncogene
**Correlation with IFI44L**						
MMP13	−0.91	−0.97	−0.71	3.86 × 10^−5^	5.38 × 10^−3^	matrix metallopeptidase 13
TFPI	−0.90	−0.97	−0.66	7.86 × 10^−5^	5.38 × 10^−3^	lipoprotein-associated coagulation inhibitor
TBP	−0.89	−0.97	−0.66	8.72 × 10^−5^	5.38 × 10^−3^	TATA box binding protein
GATA4	0.88	0.61	0.97	1.74 × 10^−4^	8.03 × 10^−3^	GATA binding protein 4
PLCB2	−0.84	−0.95	−0.52	5.71 × 10^−4^	2.11 × 10^−2^	phospholipase C, beta 2

**Table 4:** Coagulation- and fibrinolysis-related MIGs differentially expressed in PBMCs of long-term Rebif-treated versus untreated RRMS patients *.

Gene Symbol	Gene Title	*p*-Value	*p* Value (FDR)	Log2FC
**ANXA2**	**annexin A2**	**8.04** × 10^−8^	**1.06** × 10^−5^	**1.18**
ARHGEF11	Rho guanine nucleotide exchange factor 11	6.69 × 10^−6^	2.45 × 10^−4^	1.06
ARHGEF12	Rho guanine nucleotide exchange factor (GEF) 12	2.77 × 10^−3^	2.02 × 10^−2^	−1.06
ARHGEF18	Rho/Rac guanine nucleotide exchange factor 18	1.45 × 10^−4^	1.24 × 10^−2^	−1.06
ARHGEF2	Rho/Rac guanine nucleotide exchange factor 2	1.29 × 10^−4^	2.09 × 10^−3^	1.06
**EGFR**	**epidermal growth factor receptor**	**1.36 × 10^−4^**	**2.19 × 10^−3^**	**1.02**
**F13A1**	**coagulation factor XIII, A1 polypeptide**	**1.01 × 10^−2^**	**5.00 × 10^−2^**	**−1.17**
**F2R**	**coagulation factor II (thrombin) receptor**	**1.00 × 10^−2^**	**4.99 × 10^−2^**	**−1.06**
F2RL1	coagulation factor II (thrombin) receptor-like 1	3.94 × 10^−3^	2.63 × 10^−2^	1.06
F5	coagulation factor V (proaccelerin, labile factor)	3.37 × 10^−4^	4.25 × 10^−3^	1.08
GATA1	GATA binding protein 1 (globin transcription factor 1)	8.80 × 10^−3^	4.69 × 10^−2^	−1.02
GNB1	guanine nucleotide-binding protein, beta polypeptide 1	6.70 × 10^−3^	3.87 × 10^−2^	1.05
GNG5	guanine nucleotide-binding protein (G protein), gamma 5	4.89 × 10^−7^	3.69 × 10^−5^	1.10
GNG7	guanine nucleotide-binding protein (G protein), gamma 7	5.86 × 10^−5^	1.16 × 10^−3^	−1.06
GRB2	growth factor receptor-bound protein 2	1.02 × 10^−3^	9.68 × 10^−3^	1.07
**HNRNPA1**	**heterogeneous nuclear ribonucleoprotein A1**	**4.42 × 10^−5^**	**9.31 × 10^−4^**	**−1.08**
**ITPK1**	**inositol-tetrakisphosphate 1-kinase**	**2.40 × 10^−3^**	**1.81 × 10^−2^**	**1.06**
MAP2K1	mitogen-activated protein kinase kinase 1	5.03 × 10^−5^	1.03 × 10^−3^	1.05
MAPK13	mitogen-activated protein kinase 13	6.11 × 10^−3^	3.63 × 10^−2^	−1.02
MAPK14	mitogen-activated protein kinase 14	6.68 × 10^−4^	7.05 × 10^−3^	1.05
MMP13	matrix metallopeptidase 13	3.80 × 10^−3^	2.56 × 10^−2^	1.02
**NRAS**	**neuroblastoma RAS viral (v-ras) oncogene homolog**	**1.32 × 10^−3^**	**1.16 × 10^−2^**	**1.05**
**PLCB2**	**phospholipase C, beta 2**	**1.52 × 10^−6^**	**8.58 × 10^−5^**	**1.02**
PRKCA	protein kinase C, alpha	3.63 × 10^−3^	2.48 × 10^−2^	−1.07
PRKCD	protein kinase C, delta	1.56 × 10^−5^	4.38 × 10^−4^	1.15
PRKCE	protein kinase C, epsilon	4.65 × 10^−3^	2.97 × 10^−2^	1.02
PRKCI	protein kinase C, iota	3.27 × 10^−3^	2.29 × 10^−2^	1.02
PRKCZ	protein kinase C, zeta	4.33 × 10^−5^	9.15 × 10^−4^	−1.05
**RHOB**	**ras homolog family member B**	**6.69 × 10^−7^**	**4.67 × 10^−5^**	**1.33**
ROCK1	Rho-associated, coiled-coil containing protein kinase 1	1.48 × 10^−4^	2.32 × 10^−3^	1.05
**SERPINA1**	**serpin peptidase inhibitor, clade A**	**1.46 × 10^−5^**	**4.19 × 10^−4^**	**1.23**
SOS1	SOS Ras/Rac guanine nucleotide exchange factor 1	5.79 × 10^−3^	3.49 × 10^−2^	1.04
SRC	SRC proto-oncogene, non-receptor tyrosine kinase	8.21 × 10^−4^	8.23 × 10^−3^	1.06
TBP	TATA box binding protein	5.44 × 10^−3^	3.33 × 10^−2^	1.03
**THBD**	**thrombomodulin**	**2.90 × 10^−3^**	**2.10 × 10^−2^**	**1.05**

* The bold font represents genes that are common between Rebif cohort 1 and Rebif cohort 2 and have the same fold change direction in both cohorts.

## Data Availability

The dataset supporting the IFNβ-1a results, including all raw and processed microarray data and patient parameter metadata, is available in the GEO omnibus, accession number GSE73608. The LAQ arm microarray dataset analyzed in this study originates from the ALLEGRO clinical trial sponsored by Teva Pharmaceutical Industries Ltd. (Tel Aviv, Israel). Due to contractual restrictions, the raw data cannot be publicly shared. Processed gene-level results were previously published [21] and were used in the present analysis.
